# Microstructure and chemical analysis data of polyurethane-silver nanoparticles/graphene nanoplates composite fibers

**DOI:** 10.1016/j.dib.2019.104107

**Published:** 2019-06-05

**Authors:** Seung-Woo Kim, Sung-Nam Kwon, Seok-In Na

**Affiliations:** Professional Graduate School of Flexible and Printable Electronics, Department of Flexible and Printable Electronics, Polymer Materials Fusion Research Center, Chonbuk National University, Jeonju-si, 54896, South Korea

**Keywords:** Composite fiber, Microstructure and chemical analysis, Field emission scanning electron microscopy, Energy dispersive X-ray spectroscopy, Wet-spun, Silver nanoparticles, Graphene nanoplatelets

## Abstract

In this data article, we provide field emission scanning electron microscopy (FE-SEM) and energy dispersive X-ray spectroscopy (EDS) images of wet-spun polyurethane (PU)-silver nanoparticles (AgNPs)/graphene nanoplatelets (GNPs) composite fibers according to the content of AgNPs and GNPs. In addition, microstructural changes of PU-AgNPs/GNPs composite fibers due to heat treatment at various temperatures are provided. The data collected in this article is directly related to our research article “Stretchable and Electrically Conductive Polyurethane- Silver/Graphene composite fibers prepared by wet-spinning process” [1].

**Table:** Specifications table

Subject area	*Material science*
More specific subject area	*Conductive composite fiber*
Type of data	*Images (microscopy, energy dispersive X-ray spectroscopy)*
How data was acquired	*Field emission scanning electron microscopy (FE-SEM; JSM-7100F, Jeol) containing energy dispersive X-ray spectroscopy (EDS)*
Data format	*Raw and analyzed data is presented*
Experimental factors	*Long polyurethane-silver nanoparticles (AgNPs)/graphene nanoplatelets (GNPs) composite fibers with various content of AGNPs and GNPs were prepared by wet-spinning. A* 1 cm *fiber was selected randomly and observed their surface morphology and chemical composition.*
Experimental features	*FE-SEM and EDS instruments are interconnected to analyze samples simultaneously. Ag and C element mapping was done to analyze the distribution of AGNPs and GNPs.*
Data source location	*Chonbuk national university, Jeonju-si, Repubic of Korea*
Data accessibility	Data is with this article.
Related research article	*Seung-Woo Kim, Sung-Nam Kwon, Seok-In Na, Stretchable and Electrically Conductive Polyurethane- Silver/Graphene composite fibers prepared by wet-spinning process, Composite Part B: Engineering 167 (2019) 573–581;*https://doi.org/10.1016/j.compositesb.2019.03.035

**Table:** 

**Value of the data** • The data is useful to confirm the microstructural change of wet-spun composite fibers according to the content of additive. • The data is useful for analyzing the microstructural changes of composite fibers due to heat treatment and determining the proper heat treatment temperature for composite fibers. • The data is useful for analyzing the chemical element distribution of composite fibers.

## 1 Data

**Fig. 1:**
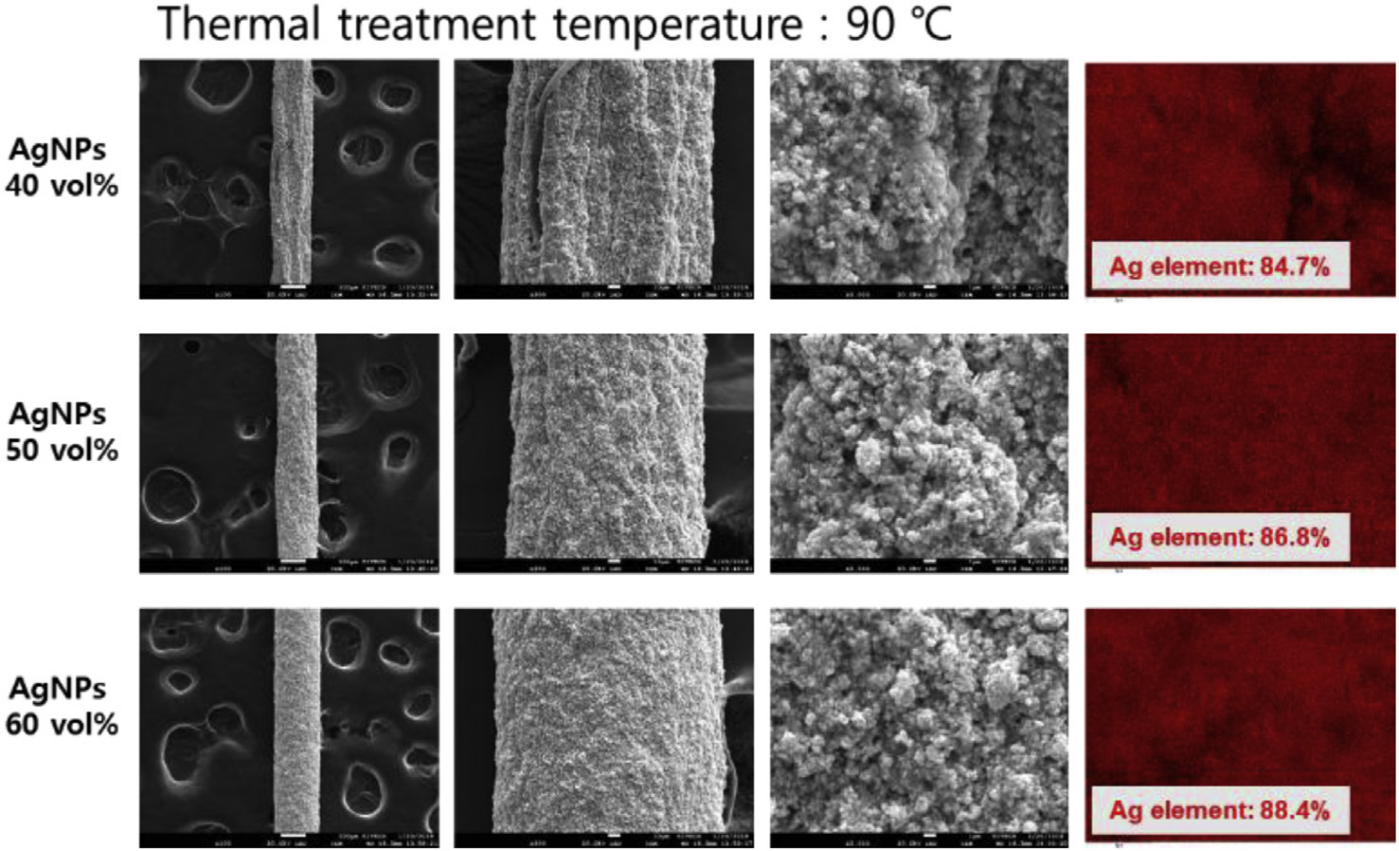
Surface FE-SEM and EDS images of the wet-spun PU-AgNPs composite fibers with the various AgNPs contents and thermal treated at 90 °C.

**Fig. 2:**
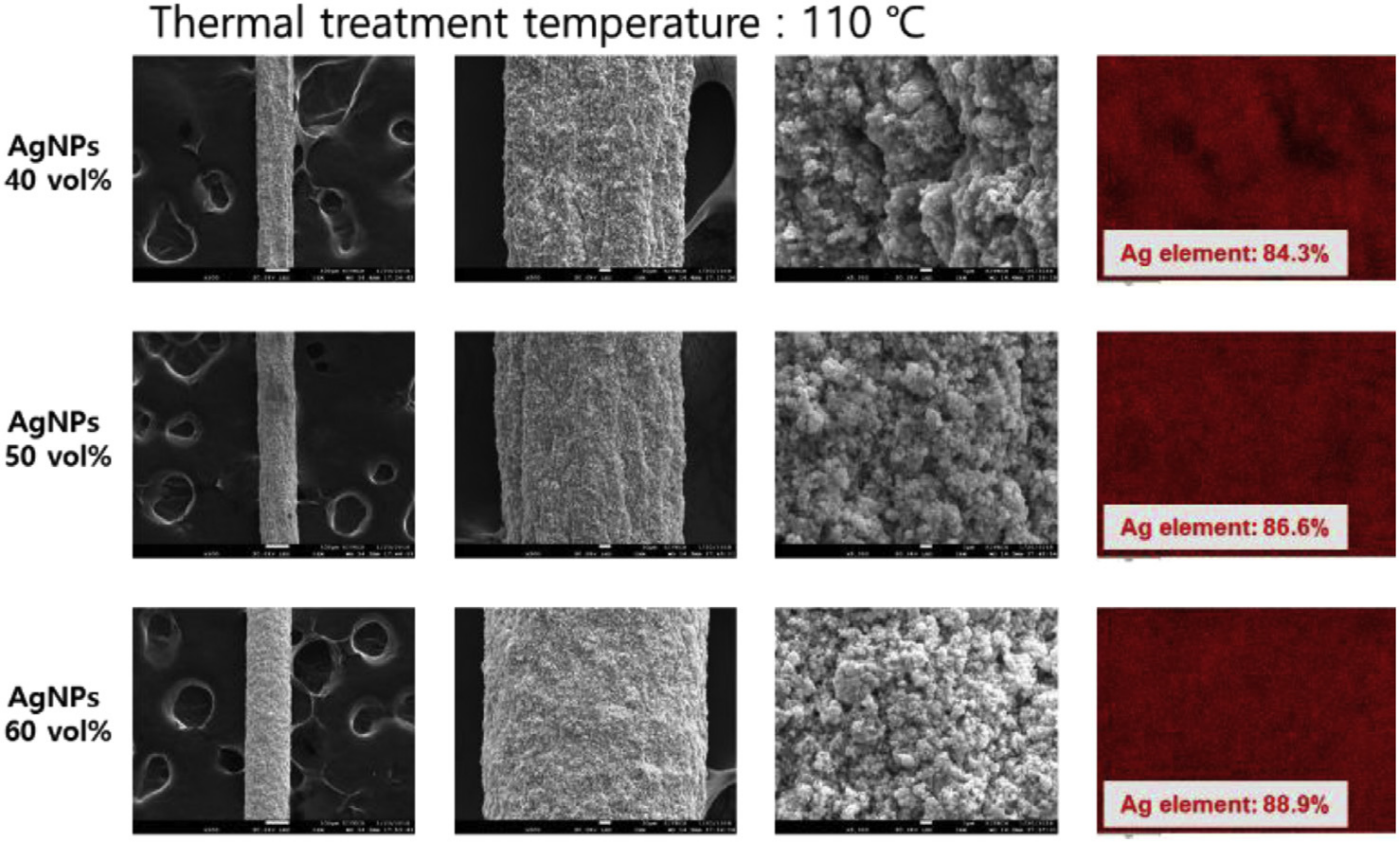
Surface FE-SEM and EDS images of the wet-spun PU-AgNPs composite fibers with the various AgNPs contents and thermal treated at 110 °C.

**Fig. 3:**
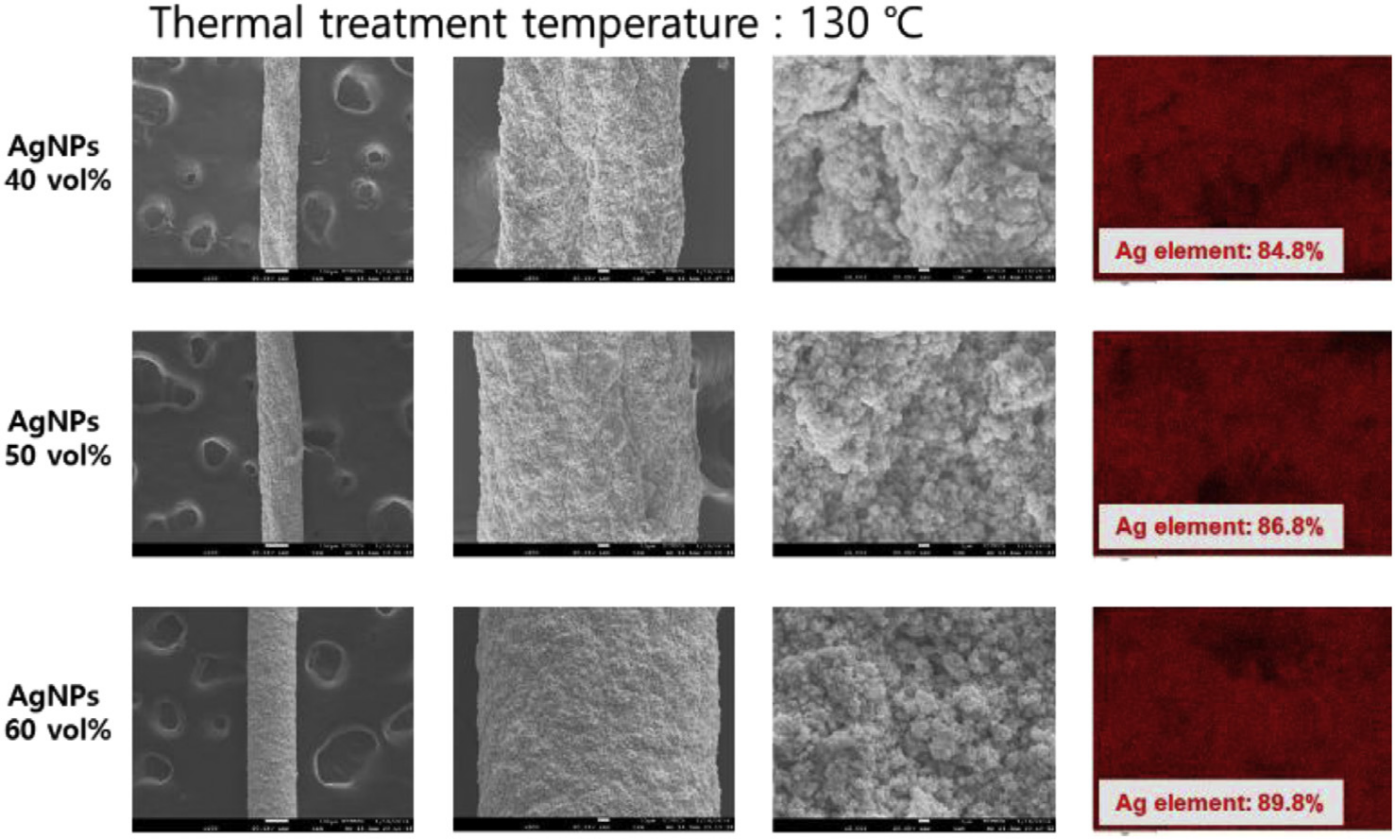
Surface FE-SEM and EDS images of the wet-spun PU-AgNPs composite fibers with the various AgNPs contents and thermal treated at 130 °C.

**Fig. 4:**
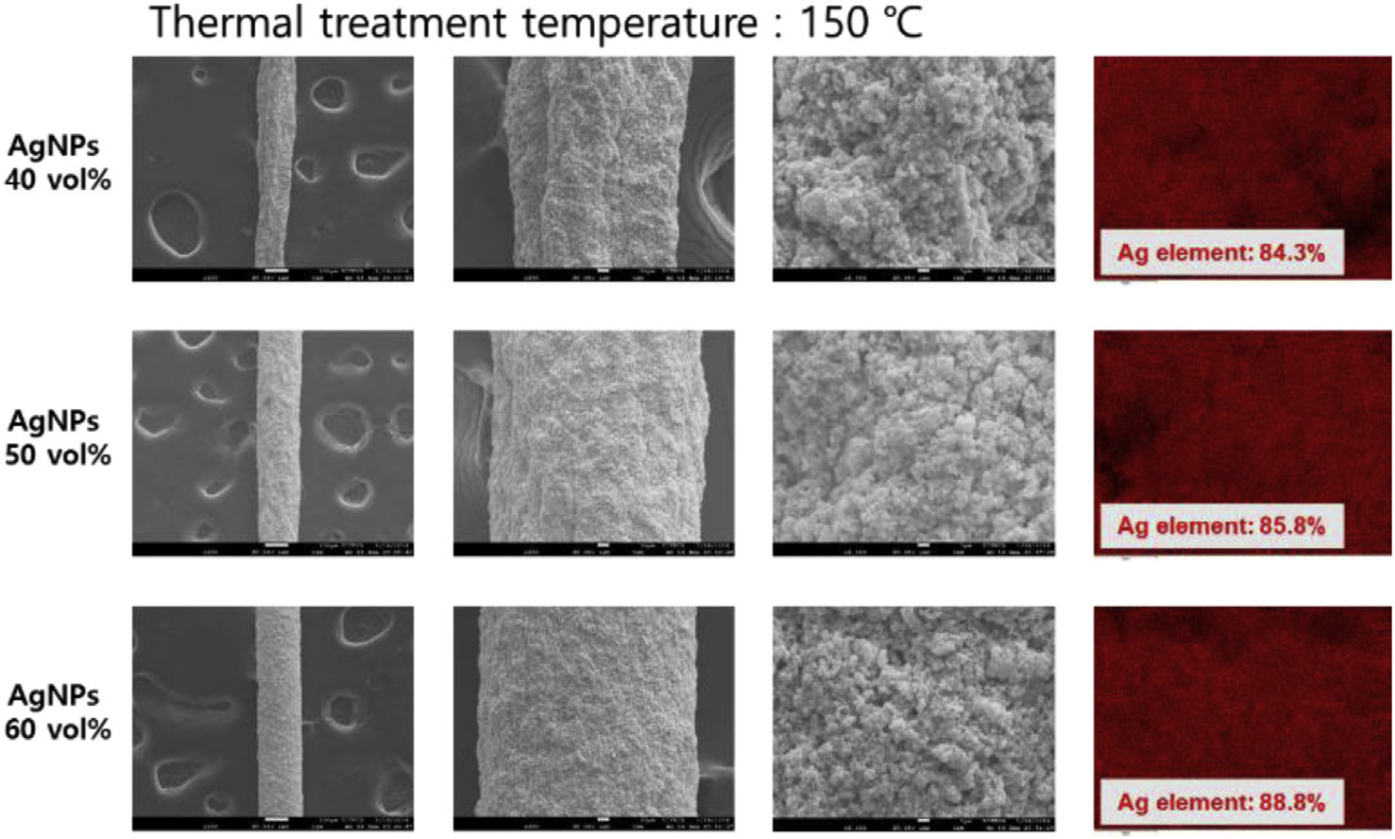
Surface FE-SEM and EDS images of the wet-spun PU-AgNPs composite fibers with the various AgNPs contents and thermal treated at 150 °C.

The data presented in this article consists of a series of FE-SEM images of PU-AgNPs/GNPs composite fibers with AgNPs and GNPs content and their thermal treated samples. In addition, surface and cross-sectional EDS images of PU-AgNPs/GNPs composite fibers were provided. As shown in Fig. 1, Fig. 2, Fig. 3, Fig. 4, we prepared the wet-spun PU-AgNPs composite fibers with 30, 40, and 50 vol% AgNPs and observed the surface microstructure changes of PU-AgNPs composite fibers with various thermal treatment temperature. It is observed that as the AgNPs content increases, the fiber shape becomes round and the fiber surface becomes smooth. In addition, it is observed that the AgNPs uniformly distributed in the PU-AgNPs composite system by EDS analysis of Ag element. The EDS chemical element mapping images of the PU-AgNPs composite fiber shows that the AgNPs were uniformly dispersed in the entire system.

**Fig. 5:**
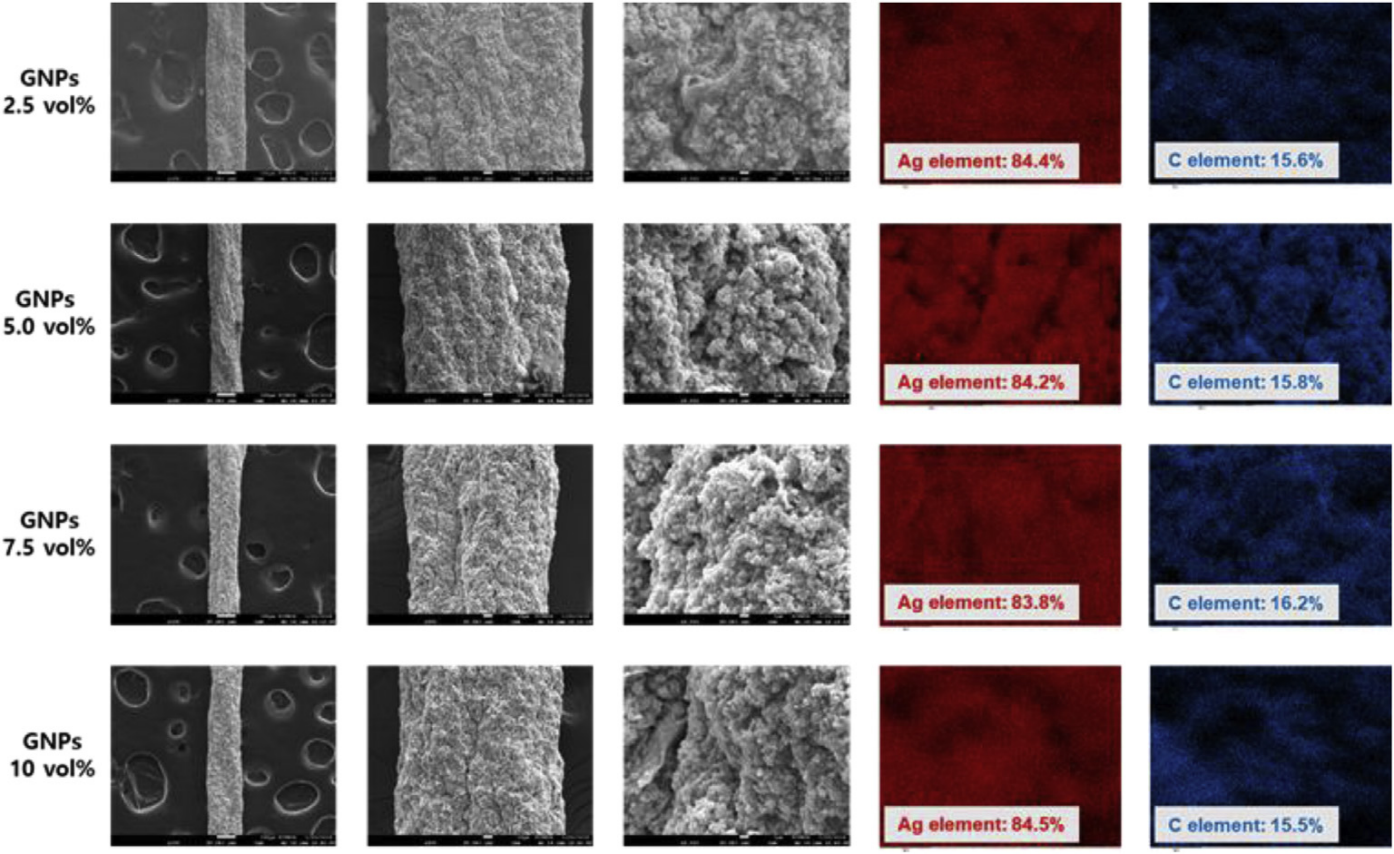
Surface FE-SEM and EDS images of the PU-AgNPs/GNPs composite fibers with 40 vol% AgNPs and 2.5, 5.0, 7.5, and 10 vol% GNPs.

Fig. 5 shows the microstructure and EDS mapping images of the wet-spun PU-AgNPs/GNPs composite fibers with 40 vol% AgNPs and 2.5, 5.0, 7.5, and 10 vol% GNPs. As shown in Fig. 5, the It is observed that the AgNPs uniformly distributed in the PU-AgNPs/GNPs composite system; the EDS chemical element mapping images of the PU-AgNPs/GNPs composite fiber confirmed that the AgNPs were uniformly dispersed in the entire system. However, GNPs is not distinguished from the PU matrix. Because C element mapping images represents carbon in both the PU matrix and GNPs, it is difficult to distinguish C signal of GNPs directly from C element mapping.

## 2 Experimental design, materials and methods

The PU-AgNPs/GNPs composite fibers were prepared by wet-spinning method [1]. And then, the PU-AgNPs/GNPs composite fibers were thermally cured at 90, 110, 130, and 150 °C for 10 minutes. In order to perform surface analysis, 1 cm of fiber was selected randomly, and the microstructure of the wet-spun PU-AgNPs and PU-AgNPs/GNPs composite fibers were examined using field emission scanning electron microscopy (FE-SEM; JSM-7100F, Jeol). The EDS chemical element mapping images were obtained simultaneously using energy dispersive X-Ray spectroscopy (EDS) interconnected with FE-SEM instrument.

## References

[1] Kim Seung-Woo, Kwon Sung-Nam, Seok-In Na (2019). Stretchable and Electrically Conductive Polyurethane- Silver/Graphene composite fibers prepared by wet-spinning process. Compos. B Eng..

